# Postpartum administration of eplerenone to mitigate vascular dysfunction in mice following a preeclampsia-like pregnancy

**DOI:** 10.1038/s41598-025-02475-0

**Published:** 2025-05-27

**Authors:** Natalie K. Binder, Natasha de Alwis, Sally Beard, Bianca R. Fato, Anjali Garg, Lydia Baird, Morag J. Young, Natalie J. Hannan

**Affiliations:** 1 Therapeutics Discovery and Vascular Function in Pregnancy Group, Department of Obstetrics and Gynaecology, The University of Melbourne and Mercy Hospital for Women, Heidelberg, VIC 3084 Australia; 2 Cardiovascular Endocrinology Laboratory, Baker Heart and Diabetes Institute, Prahran, VIC 3181 Australia; 3 Baker Department of Cardiometabolic Health, Melbourne Medical School, The University of Melbourne, Parkville, VIC 3010 Australia

**Keywords:** Pregnancy, Preeclampsia, Eplerenone, Mouse model, L-NAME, Cardiovascular disease, Cardiology, Diseases, Medical research

## Abstract

Preeclampsia is a severe pregnancy complication associated with substantial injury to systemic vasculature, major organs, and the feto-placental unit, with an approximate mortality rate of 76,000 pregnant women and 500,000 babies each year. Preeclampsia results in up to five-fold increased risk of cardiovascular disease. There is currently no cure and limited treatment options for preeclampsia and its long-term effects. In this study, we modelled preeclampsia in the mouse via nitric oxide blockade and examined the effect of therapeutic intervention during pregnancy (esomeprazole) and postpartum (eplerenone) on indices of cardiovascular health. Pregnant CBA x C57BL/6 mice received 50 mg/kg/day N(ω)-nitro-L-arginine methyl ester to induce a preeclampsia-like phenotype. Mice were treated with either 12.5 mg/kg/day esomeprazole during pregnancy, 55.5 mg/kg/day eplerenone during the postpartum period, or both esomeprazole and eplerenone in sequence. Mice were hypertensive during pregnancy, fetal growth was restricted by 10%, and maternal vasoactivity was impaired at 5-weeks postpartum. Eplerenone treatment (± esomeprazole) reduced vasoconstriction at 5-weeks postpartum and enhanced vasorelaxation at 5- and 10-weeks postpartum, supporting improved cardiovascular indices in the medium to long term postpartum period. Postpartum eplerenone treatment may be beneficial in mitigating consequent cardiovascular disease risk following a pregnancy complicated by preeclampsia.

**Supplementary Information:**

The online version contains supplementary material available at 10.1038/s41598-025-02475-0.

## Introduction

Preeclampsia is a severe obstetric disease that impacts 2–8% of pregnancies worldwide^1–3^. The aetiology of preeclampsia is likely attributed to ischemia-reperfusion injury of the placenta, leading to a hypoxic environment and release of anti-angiogenic and pro-inflammatory factors into maternal circulation^4–7^. Systemic endothelial dysfunction ensues and contributes to the clinical manifestations of preeclampsia such as *de-novo* hypertension, systemic vasoconstriction, and reduced organ perfusion^8,9^. Preeclampsia is associated with significant maternal and fetal morbidity and mortality during pregnancy, and with endothelial dysfunction shown to persist postpartum^10^, long-term health outcomes are also impacted for both mother and child^11^. Following delivery, these individuals are at a significantly higher risk of cardiovascular sequalae than those with normotensive pregnancies, even after adjustment for confounding risk factors^12–17^.

Identifying treatments for preeclampsia and its associated cardiovascular risks is limited by an incomplete understanding of disease pathophysiology. While drugs such as aspirin show promise in alleviating disease risk prophylactically^18,19^, there are currently limited therapies to arrest disease progression^20^. Delivery of the baby and the dysfunctional placenta is the only ‘cure’ for preeclampsia but imposes morbidity risks for the infant when done prematurely^21^. Hence, there is a need to identify therapeutics that directly target the disease and reduce long-term cardiovascular risk^22^.

Mineralocorticoid receptor (MR) antagonists such as eplerenone (EPL) reduce hypertensive phenotypes and improve cardiovascular outcomes in all cause heart failure and other forms of heart and vascular disease^23,24^. EPL is selective for the MR and has established cardiac and vascular protective effects including reduced vessel wall remodelling and cardiac inflammation, and reduced fibrosis and dysfunction^25,26^, which are hallmarks of postpartum cardiovascular dysfunction following preeclampsia. In epithelial tissues such as the distal nephron, EPL blocks the actions of aldosterone, which is a downstream effector hormone of the renin-angiotensin-aldosterone system (RAAS)^27^. Aldosterone is a key effector of fluid volume, blood pressure, and acid-base balance, regulating ion channels within the renal epithelium^28^. During pregnancy, elevated aldosterone levels follow a fall in vascular resistance, insensitivity to vasoactive hormones, changes in sodium excretion, and elevated progesterone levels, and are thus an important regulator of the increased blood volume required to support pregnancy^29^. However, in preeclampsia, aldosterone levels are often paradoxically lower or inappropriately suppressed despite the presence of hypertension^30,31^. This is likely due to dysregulation of RAAS, increased natriuretic peptides, and elevated anti-angiogenic factors such as soluble fms-like tyrosine kinase-1. Less is known about postpartum aldosterone levels following a pregnancy complicated by preeclampsia. Aldosterone also influences sympathetic activity and inflammatory processes due to MR activation in non-epithelial tissues such as the brain, heart, vasculature, and adipose tissue^32,33^. Dysregulated aldosterone and inappropriate activation of the MR are associated with undesirable cardiovascular pathology^34–36^. EPL and other MR antagonists reduce atherosclerotic lesions, hypertension, and oxidative stress in mice^37,38^ and in the clinic^39^. However, to date, no studies have investigated the role of EPL for cardio-protection following hypertensive pregnancy disorders such as preeclampsia.

Proton pump inhibitors (PPIs) such as esomeprazole (ESO) are a class of drugs used for gastro-intestinal complications such as gastric reflux and peptic ulcers^40^. PPIs are deemed safe during pregnancy^41,42^ and emerging evidence suggests they could be repurposed to treat preeclampsia^20,43^. In vitro, PPIs decrease the release of anti-angiogenic and pro-inflammatory factors from placental cells and tissue, as well as decrease blood pressure in rat and mouse models of gestational hypertension^44,45^. Collectively, this suggests that ESO may be able to modify key drivers of preeclampsia pathophysiology, particularly through reduced endothelial dysfunction and circulating inflammatory and anti-angiogenic mediators, and enhanced cytoprotection and vasorelaxation. An initial clinical trial of esomeprazole in South African preeclamptic women did not show significant clinical improvement^46^. However, this trial was likely underpowered and required higher dosage as only low levels of drug were detected in maternal circulation after administration.

This study aimed to examine the effects of postpartum EPL treatment on markers of cardiovascular health following a preeclampsia-like pregnancy in mice, with or without ESO treatment during pregnancy (which has shown efficacy previously^44^.

## Methods

### Animal studies

All animal experiments were approved by the Austin Health Animal Ethics Committee (A2018/05596) and followed the National Health and Medical Research Council ethical guidelines for the care and use of animals for scientific purposes. This manuscript complies with the ARRIVE guidelines for the reporting of in vivo experiments. Four- to 8-week-old CBA x C57BL/6 (F1) female mice (*n* = 78) from Animal Resources Centre (Western Australia, Australia) were housed at the BioResources Facility, Austin Health Heidelberg Campus. Mice were group-housed in conventional open-top cages, on a 12-h light/dark cycle, with food and water ad libitum (18–22 °C; 50% relative humidity). Prior to experimentation, mice were acclimated to the CODA non-invasive blood pressure system (Kent Scientific, Torrington, CT, USA) in a seven-stage process involving repeated exposure to the restraining tube and tail cuff^47,48^.

Reproductively mature adult mice were used in the current study to avoid potential confounding factors associated with peri-pubertal mice or mice approaching reproductive senescence. From 8 to 14 weeks of age, F1 females were mated overnight with F1 male mice. Pregnancy was confirmed by the presence of a copulatory plug the following morning, designated as embryonic day (E)0.5, at which point the mice were randomly assigned to an experimental group (Table 1).

**Table 1:** Experimental groups.

	Pregnancy	Postpartum
Normal	PBS + vehicle	Vehicle
Vehicle	L-NAME + vehicle	Vehicle
ESO	L-NAME + ESO	Vehicle
ESO + EPL	L-NAME + ESO	EPL
EPL	L-NAME + vehicle	EPL

Phosphate buffered saline (PBS), N(ω)-nitro-L-arginine methyl ester (L-NAME), esomeprazole (ESO), eplerenone (EPL).

### Mouse model of preeclampsia

Administration of N(ω)-nitro-L-arginine methyl ester (L-NAME) to mice during pregnancy increases maternal blood pressure and causes fetal growth restriction^49^, a phenotype consistent with the clinical characterisation of preeclampsia^50^. L-NAME (50 mg/kg/day; Sigma-Aldrich, St. Louis, MO, USA) was administered daily from E7.5 to E18.5 (parturition occurs during the dark cycle following E18.5) via 100 µL subcutaneous injection, along with 0.9% NaCl drinking water. Immediately following L-NAME injection, mice received either a 100 µl intraperitoneal injection of ESO sodium (12.5 mg/kg/day; Abcam, Cambridge, UK) in phosphate buffered saline (PBS; Life Technologies, Carlsbad, USA) or PBS as a vehicle control. Both injections were administered while the mouse remained restrained, without releasing it between doses. This approach minimised handling time and stress by reducing the number of restraint events per dosing session. Normal pregnant mice (non-hypertensive) received sham L-NAME injections (100 µl PBS, subcutaneous) and ESO vehicle injections (100 µl PBS, intraperitoneal). On E13.5, E15.5, and E17.5 of pregnancy, blood pressure was measured.

On day 1 postpartum, neonates were counted, weighed, and had crown-to-rump length measured before being culled. From 1 week postpartum, dams received either EPL (55 mg/kg/day; Sapphire Biosciences, New South Wales, Australia) via voluntary oral administration in peanut butter (100 mg), or peanut butter as a vehicle control. Maternal blood pressure was measured every week postpartum for up to 10 weeks. A subset of mice (50%) were allocated to postpartum week (PP)5 endpoint, with the remainder having a PP10 endpoint. At endpoint, urine was collected by scruff-induced bladder voiding prior to anaesthesia with 5% isoflurane in oxygen for blood collection via cardiac puncture. Mice were then culled by cervical dislocation (PP5 average weight at cull 24.8 g, PP10 average weight at cull 26.7 g). Blood samples were allowed to coagulate at room temperature before centrifugation to separate the serum fraction, which was snap frozen and stored at − 80 °C.

Maternal hearts and kidneys were fixed in 4% paraformaldehyde (FUJIFILM Wako Chemicals, Richmond, USA) for 5 h followed by storage in 70% ethanol until processing, or preserved in RNAlater (Invitrogen, Carlsbad, USA) for a minimum of 48 h before being snap frozen and stored at -80 °C until analysis. The intestinal tract was collected in ice cold PBS for dissection of mesenteric arteries for vascular studies^48,51^.

### Vascular reactivity studies

Second order mesenteric arteries were dissected from surrounding connective and adipose tissue in Krebs physiological salt solution (NaCl 120 mM, KCl 5 mM, MgSO_4_ 1.2 mM, KH_2_PO_4_ 1.2 mM, NaHCO_3_ 25 mM, D-glucose 11.1 mM, CaCl_2_ 2.5 mM). Dissected arteries (2 mm length) were mounted on the 620 M Wire Myograph (Danish Myo Technology (DMT), Hinnerup, Denmark) using 25 μm diameter gold-plated tungsten wires (W005230; Goodfellow, Cambridge, England), and bathed in Krebs solution continuously bubbled with carbogen (95% O_2_, 5% CO_2_) at 37 °C. Arteries were normalised to 100 mmHg (13.3 kPa) pressure using the DMT normalisation module on LabChart software (ADInstruments, Sydney, NSW, Australia) with IC1/1C100 = 1. Smooth muscle viability was confirmed using high potassium physiological salt solution (KPSS; NaCl 25 mM, KCl 100 mM, MgSO_4_ 1.2 mM, KH_2_PO_4_ 1.0 mM, NaHCO_3_ 25 mM, D-glucose 11.1 mM, CaCl_2_ 2.5 mM). Endothelial function was assessed by pre-constricting arteries to 50–70% of maximal constriction to KPSS with phenylephrine (Sigma-Aldrich), then relaxed with endothelial-dependent dilator, acetylcholine (Sigma-Aldrich). Greater than 80% relaxation was required for vessel inclusion. Constriction and relaxation dose response curves were then generated using phenylephrine and acetylcholine (10^− 9^ to 10^− 4.5^ M)^47,48,51,52^. Relaxation dose response curves were generated by pre-constricting the arteries to 50–70% of maximal constriction to KPSS, followed by cumulative addition of acetylcholine doses as previously described^47,48,51,52^ and in accordance with the guidelines for the measurement of vascular function and structure in isolated arteries and veins^53^. The response at each dose was allowed to plateau before addition of the next dose.

### Quantitative polymerase chain reaction (qPCR)

RNA was extracted from RNAlater preserved hearts and kidneys using the GenElute™ Mammalian Total RNA Miniprep Kit (Sigma-Aldrich) and quantified with a Nanodrop 2000 spectrophotometer (ThermoFisher Scientific, Waltham, MA, USA). Total RNA was converted to cDNA using the Applied Biosystems^TM^ High-Capacity cDNA Reverse Transcription Kit, as per manufacturer guidelines on the iCycler iQ5 (Bio-Rad, Hercules, CA, USA).

Quantitative PCR with Taqman reagents was performed to quantify RNA expression using primers purchased from Life Technologies. Primers used were designed for: Vascular cell adhesion protein-1 (*Vcam1* Mm01320970_m1), transforming growth factor beta 1, 2, and 3 (*Tgfb1* Mm01178820_m1, *Tgfb2* Mm00436955_m1, and *Tgfb3* Mm00436960_m1, respectively), matrix metallopeptidase 2 and 9 (*Mmp2* Mm00439498_m1 and *Mmp9* Mm00442991_m1), C-C motif chemokine receptor 2 (*Ccr2* Mm04207877_m1), calcium/calmodulin-dependent protein kinase II alpha (*Cam2ka* Mm00437967_m1), natriuretic peptide type B (*Bnp* Mm01255770_g1), connective tissue growth factor (*Ctgf* Mm01192933_g1), endothelin-1 (*Edn1* Mm00438659_m1), NADPH oxidase 2 (*Nox2* Mm01287743_m1), TIMP metallopeptidase inhibitor 1 (*Timp1* Mm01341361_m1), nuclear receptor subfamily 3 group C member 1 (*Nr3c1* Mm00433832_m1), nuclear receptor subfamily 3 group C member 2 (*Nr3c2* Mm01241596_m1), solute carrier family 9 member A1 (*Slc9a1* Mm00444270_m1), serum/glucocorticoid regulated kinase 1 (*Sgk1* Mm00441380_m1), sodium channel epithelial 1 subunit alpha (*Scnn1a* Mm00803386_m1), hydroxysteroid 11-beta dehydrogenase 2 (*Hsd11b2* Mm01251104_m1), NADPH oxidase 4 (*Nox4* Mm00479246_m1), fibronectin 1 (*Fn1* Mm01256744_m1), and reference gene ubiquitin C (*Ubc* Mm01198158_m1).

Quantitative PCR was performed on the CFX384 (Biorad) with run conditions: 50 °C for 2 min; 95 °C for 20 s, 95 °C for 3 s, 60 °C for 30 s (40 cycles). Expression data were normalized to expression of reference gene as an internal control and calibrated against the average Ct of the control samples, with each biological sample run in technical duplicate.

### Enzyme linked immunosorbent assay (ELISA)

Concentrations of endothelin 1 (ET-1) and C-Reactive Protein (CRP) in dam serum were measured by Mouse Endothelin-1 Quantikine ELISA Kit and Mouse C-Reactive Protein/CRP Quantikine ELISA Kit (R&D Systems, Minneapolis, MN, USA), respectively, according to manufacturer instructions.

### Urine albumin to creatinine assessment

Albumin and creatinine concentrations in dam urine were measured by Mouse Albumin ELISA kit (ab108792; Abcam) and Mouse Creatinine Kit (ab65340; Abcam), respectively, run according to manufacturer instructions.

### Maternal heart and kidney histology

Following fixation, paraffin embedded tissue was sectioned on a microtome at 4 μm thickness. Sections were deparaffinized in xylene and rehydrated through descending grades of ethanol. Cardiac collagen content was evaluated in dewaxed sections stained with 0.1% Sirius red for 30 min (Sigma-Aldrich), followed by 2 rinses in tap water, rapid dehydration, and mounting in Depex. Whole sections were scanned using the Aperio Scanscope AT Turbo (Leica Biosystems, Germany; Monash University Histology Platform). Evaluation of images for relative collagen content was performed as described previously using ImageJ where the investigator was blinded to sample IDs^54^. Representative images are derived from Aperio scans.

Cardiac and renal immunohistochemistry for Mac2 + monocytes/macrophages was performed using rat anti-mouse galectin 3 antibody (R&D Systems; 1:100 overnight at 4 °C) with rabbit anti-rat secondary (Vector Labs; 1:200, 1 h room temperature). Representative images were acquired at 40x magnification (Olympus BX43 Microscope)^54,55^.

### Statistical analysis

Data was assessed for normal (Gaussian) distribution and differences between groups at each time point were statistically tested non-parametrically with a Mann–Whitney test or parametrically with a One-way ANOVA, as appropriate. Nested One-way ANOVA was used for analysis of fetal data, with dams being experimental replicates and individual fetuses being technical replicates. Myograph dose-response curves were produced using a non-linear regression analysis (log[agonist] vs. response – four parameters). Comparison of responses to the agonist were tested for significance using Mixed-effects analysis, with Šidák correction for multiple comparisons. P-values < 0.05 were considered significantly different. Statistical analysis was performed using GraphPad Prism 10 software (La Jolla, CA, USA).

## Results

### L-NAME induces a preeclampsia-like pregnancy in mice

**Fig. 1:**
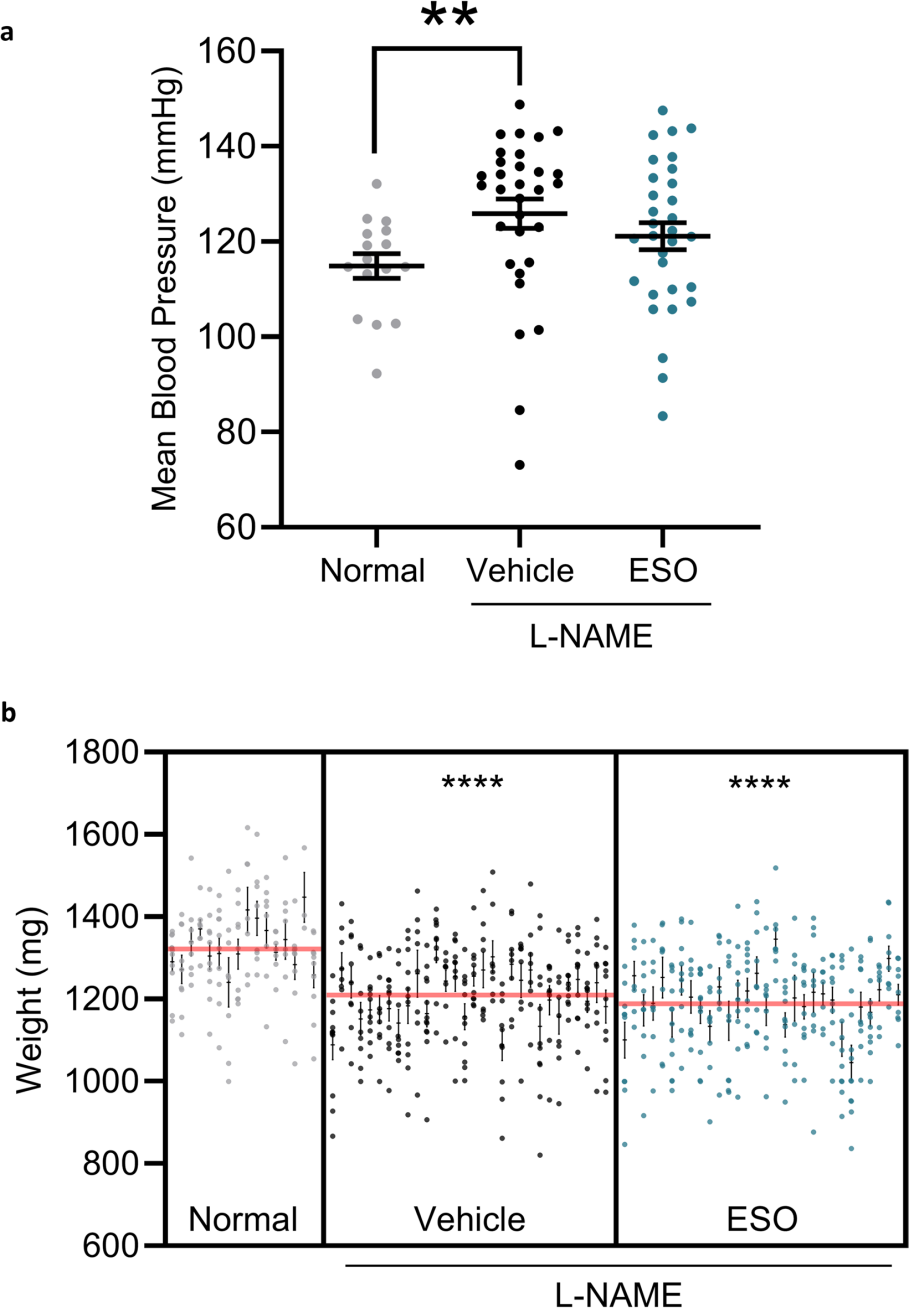
L-NAME induced a preeclampsia-like pregnancy. On gestational day 17.5, mean arterial blood pressure was significantly higher in mice receiving L-NAME (vehicle) compared to normal pregnant mice (normal) (**a**). The blood pressure of mice receiving L-NAME and treated with esomeprazole (ESO) was not significantly different from vehicle or normal pregnant. Pup weight at birth was significantly lower from mice receiving L-NAME, with or without esomeprazole treatment, compared with pups from normal pregnant mice (**b**). Data are mean ± SEM, *n* = 16–31, **p < 0.01, ****p < 0.0001.

L-NAME administration during pregnancy induced a preeclampsia-like phenotype. Mice receiving L-NAME had significantly higher mean arterial blood pressure on E17.5 of pregnancy compared to normal pregnant mice (125.9 ± 3.1 mmHg vs. 114.9 ± 2.6 mmHg, respectively, *p* < 0.01; Fig. 1). ESO treatment of mice receiving L-NAME did not significantly reduce blood pressure (121 ± 2.8 mmHg). Blood pressure measured at E13.5 and E15.5 was not different between the groups (data not shown).

Pups born to dams receiving L-NAME during pregnancy had significantly lower birth weights than pups from normal pregnant dams (1206 ± 7 mg vs. 1320 ± 10 mg, respectively, *p* < 0.0001; Fig. 1). Birth weights of pups born to dams receiving L-NAME and ESO during pregnancy (1189 ± 8 mg) were equivalent to mice receiving L-NAME alone.

### Postpartum blood pressure was not different following a preeclampsia-like pregnancy

**Fig. 2:**
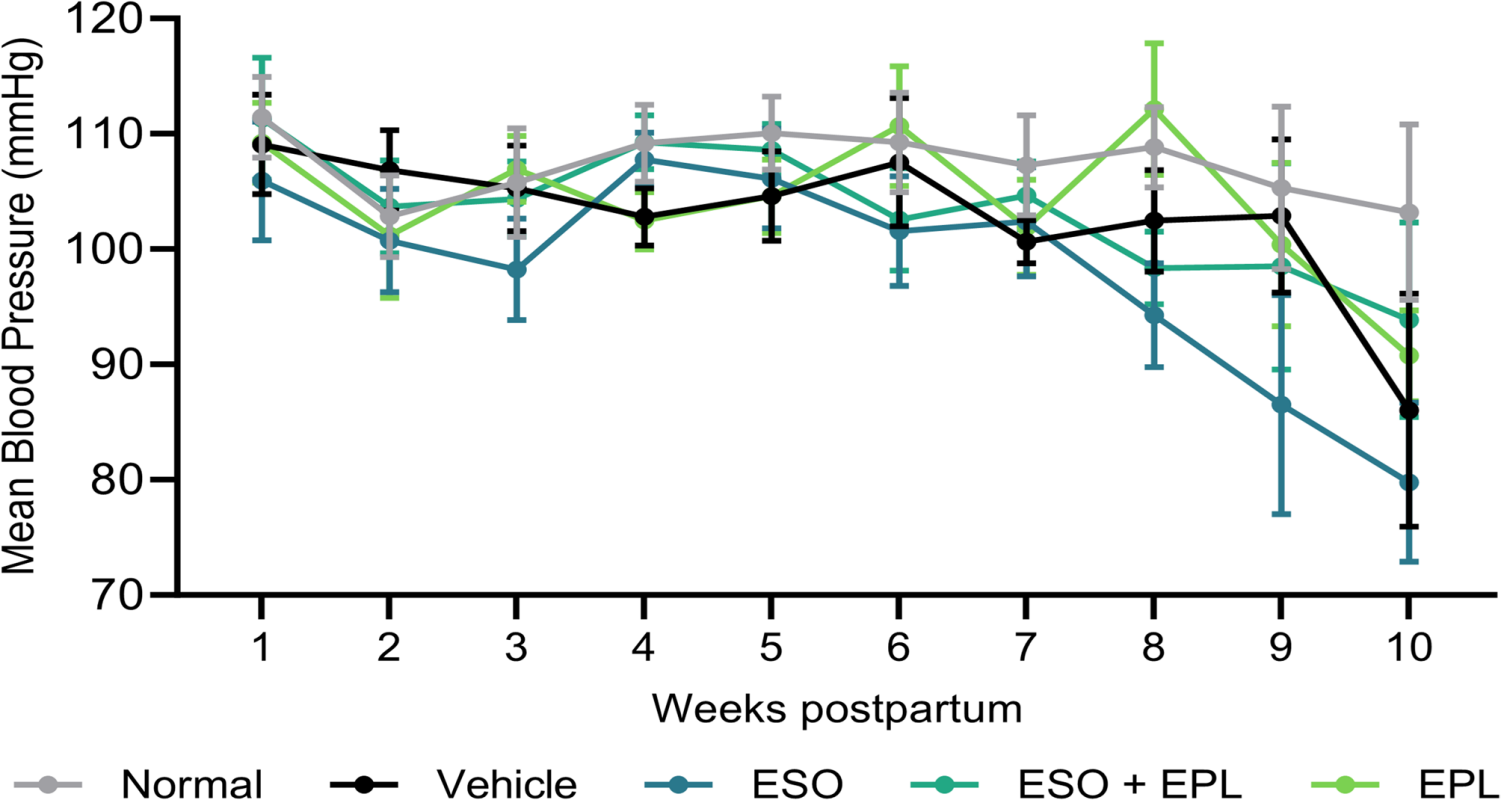
A preeclampsia-like pregnancy in mice did not affect postpartum blood pressure. Measured weekly from the time of littering until 10 weeks postpartum, mean arterial blood pressure did not differ significantly between mice that had a preeclampsia-like pregnancy (vehicle) and those that had a normal pregnancy (normal). Treatment with esomeprazole (ESO) during pregnancy, eplerenone (EPL) during the postpartum period, or both therapeutics sequentially (ESO + EPL) did not affect postpartum blood pressure either. Data are mean ± SEM.

Postpartum blood pressure, measured weekly from 1 to 10 weeks postpartum, did not differ significantly between mice that had a preeclampsia-like pregnancy and those that had a normal pregnancy (Fig. 2). For mice that had a preeclampsia-like pregnancy, treatment with ESO, EPL, or sequential ESO + EPL did not affect postpartum blood pressure.

### Postpartum vascular constriction was increased following a preeclampsia-like pregnancy and improved with postpartum EPL treatment

**Fig. 3:**
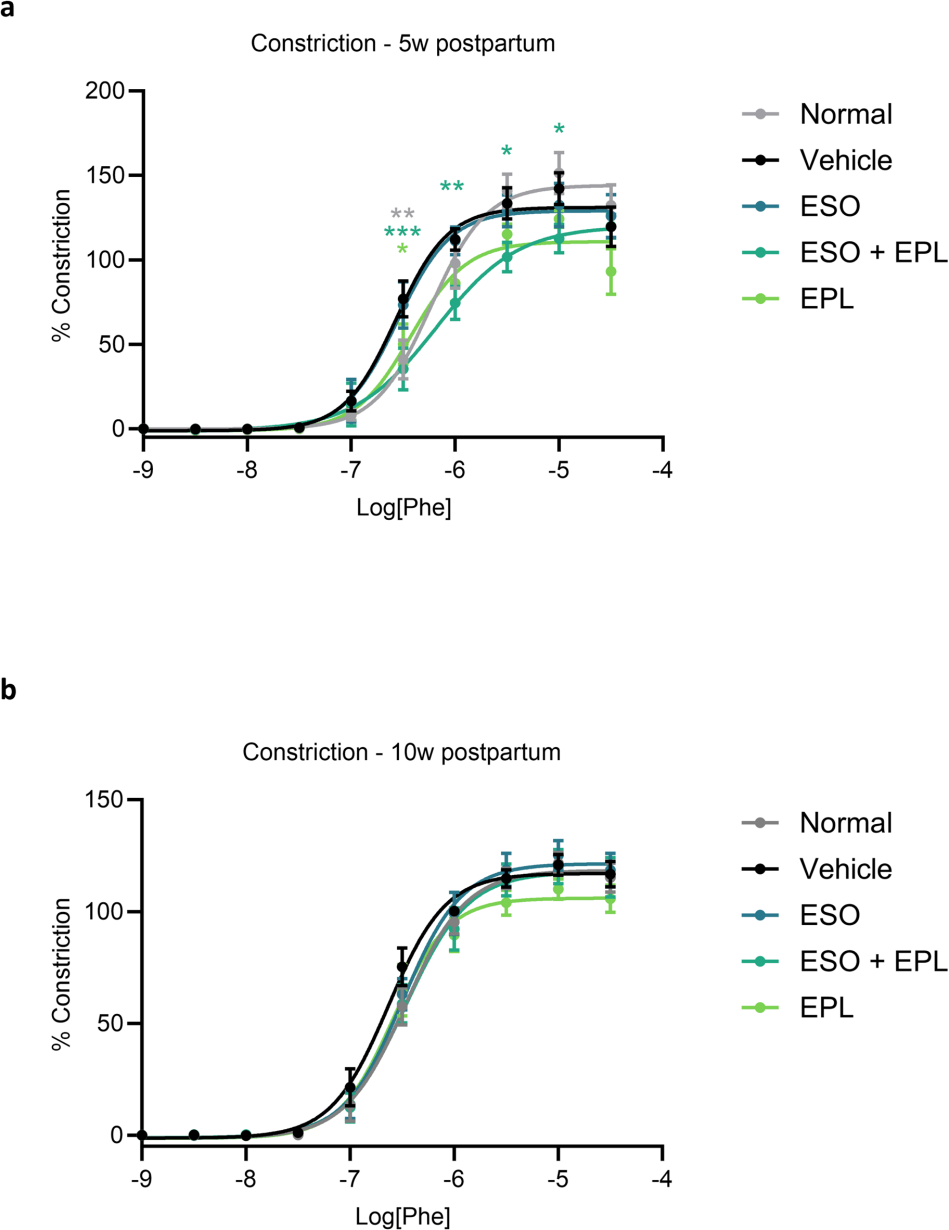
Sequential esomeprazole and eplerenone treatment decreases vasoconstriction. Excised mesenteric arteries from mice that had a preeclampsia-like pregnancy (vehicle) had increased vasoconstriction to phenylephrine at 5 weeks postpartum compared to mice that had a normal pregnancy (normal) (**a**). Sequential treatment with esomeprazole during pregnancy and eplerenone during the postpartum period (ESO + EPL) significantly reduced vasoconstriction. Eplerenone alone (EPL) had a moderate reduction in vasoconstriction, while esomeprazole alone (ESO) had no effect. No significant differences were observed at 10 weeks postpartum (**b**). Constriction response to phenylephrine was normalised to the maximal constriction to high potassium solution (KPSS). Data are mean ± SEM, *n* = 6–8, *p < 0.05, **p < 0.01, ***p < 0.001.

Mice who had a preeclampsia-like pregnancy showed increased vasoconstriction of mesenteric arteries to one dose of phenylephrine (10^− 6.5^ M) ex vivo at 5 weeks postpartum, compared to mice that had a normal pregnancy (*p* < 0.01; Fig. 3). For mice that had a preeclampsia-like pregnancy, sequential ESO + EPL treatment significantly reduced vasoconstriction compared to mice that had a preeclampsia-like pregnancy without treatment, at phenylephrine doses 10^− 6.5^–10^− 5^ M (*p* < 0.05–0.001; Fig. 3). EPL alone also reduced vasoconstriction, reaching significance at 10^− 6.5^ M phenylephrine (*p* < 0.05), while ESO alone did not affect vasoconstriction. No significant changes were observed in the LogEC50 (half-maximal response corresponding to the shift of the curve) or maximum response of vasoconstriction (Supplementary Fig. 1), but the area under the curve (total response) for sequential ESO + EPL treatment was significantly reduced compared to mice who had a preeclampsia-like pregnancy without treatment (*p* < 0.05).

At 10 weeks postpartum, ex vivo vasoconstriction of mesenteric arteries to phenylephrine had recovered and was not significantly different between mice that had a normal pregnancy or a preeclampsia-like pregnancy, with or without ESO and/or EPL treatment (Fig. 3 and Supplementary Fig. 1).

### Postpartum vascular relaxation was increased with EPL treatment

**Fig. 4:**
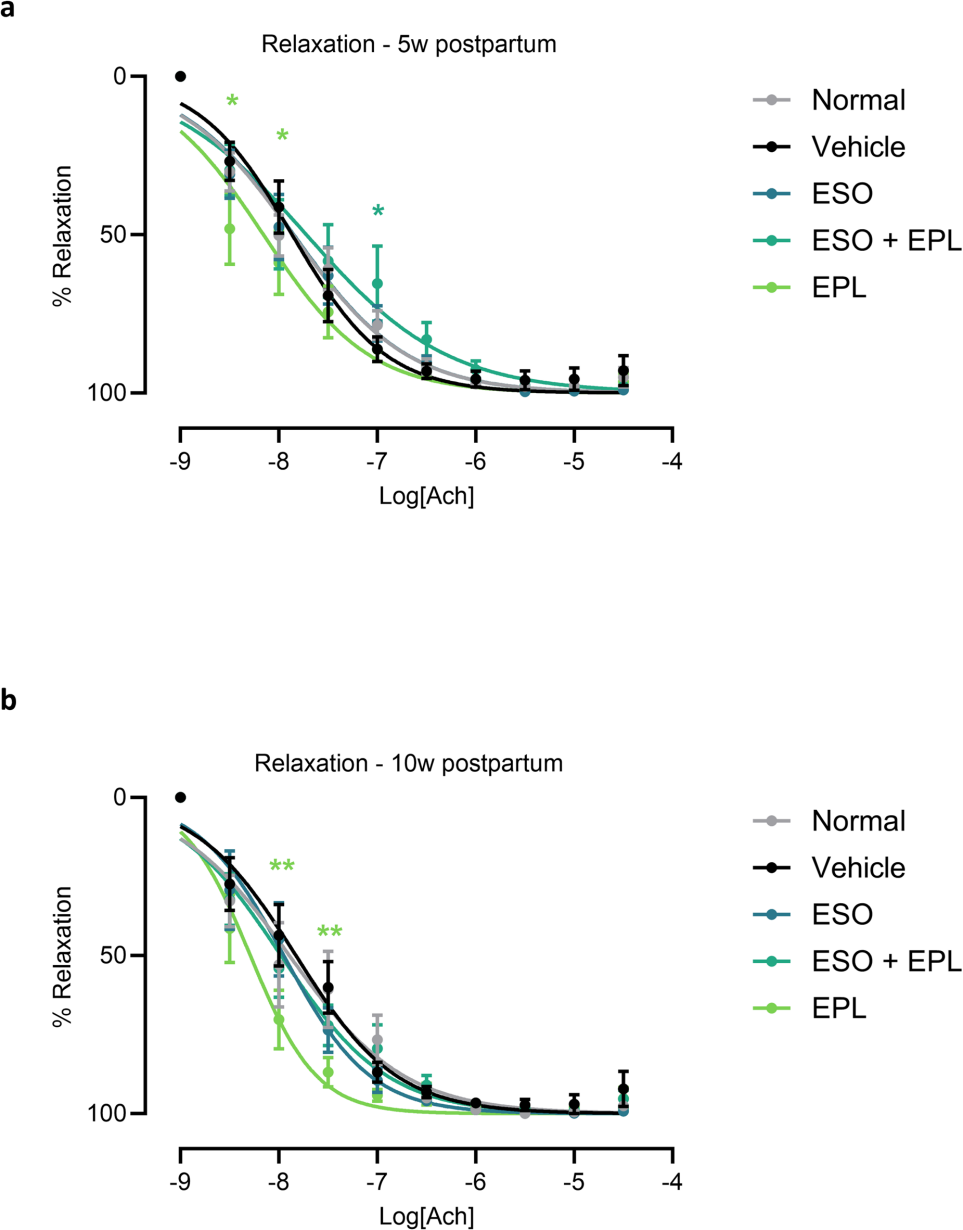
Postpartum eplerenone treatment increases vasorelaxation. There was no difference in vasorelaxation to acetylcholine in excised mesenteric arteries from mice that had a preeclampsia-like pregnancy (vehicle) compared to mice that had a normal pregnancy (normal) at 5 (**a**) and 10 (**b**) weeks postpartum. At both timepoints, eplerenone (EPL) significantly increased vasorelaxation, esomeprazole (ESO) had no effect, and sequential esomeprazole and eplerenone (EOS + EPL) reduced vasorelaxation at one dose of acetylcholine. Data are mean ± SEM, *n* = 6–8, *p < 0.05, **p < 0.01.

Compared to mice that had a normal pregnancy, ex vivo vasorelaxation of mesenteric arteries to acetylcholine at both 5 and 10 weeks postpartum was not different in mice who had a preeclampsia-like pregnancy (Fig. 4). Despite no change with a preeclampsia-like pregnancy, postpartum EPL treatment significantly enhanced vasorelaxation at both 5 and 10 weeks postpartum at acetylcholine doses 10^− 8.5^–10^− 8^ M (*p* < 0.05) and 10^− 8^–10^− 7.5^ M (*p* < 0.01), respectively. Sequential ESO + EPL treatment significantly reduced vasorelaxation at 10^− 7^ M acetylcholine at 5 weeks postpartum (*p* < 0.05), but was without effect at 10 weeks postpartum (Fig. 4). ESO treatment alone did not affect vasorelaxation. There was no change in LogEC50, area under the curve, or maximum relaxation at either postpartum timepoint (Supplementary Fig. 2).

### Circulating indicators of cardiovascular risk are not elevated 5 and 10 weeks postpartum following a preeclampsia-like pregnancy

**Fig. 5:**
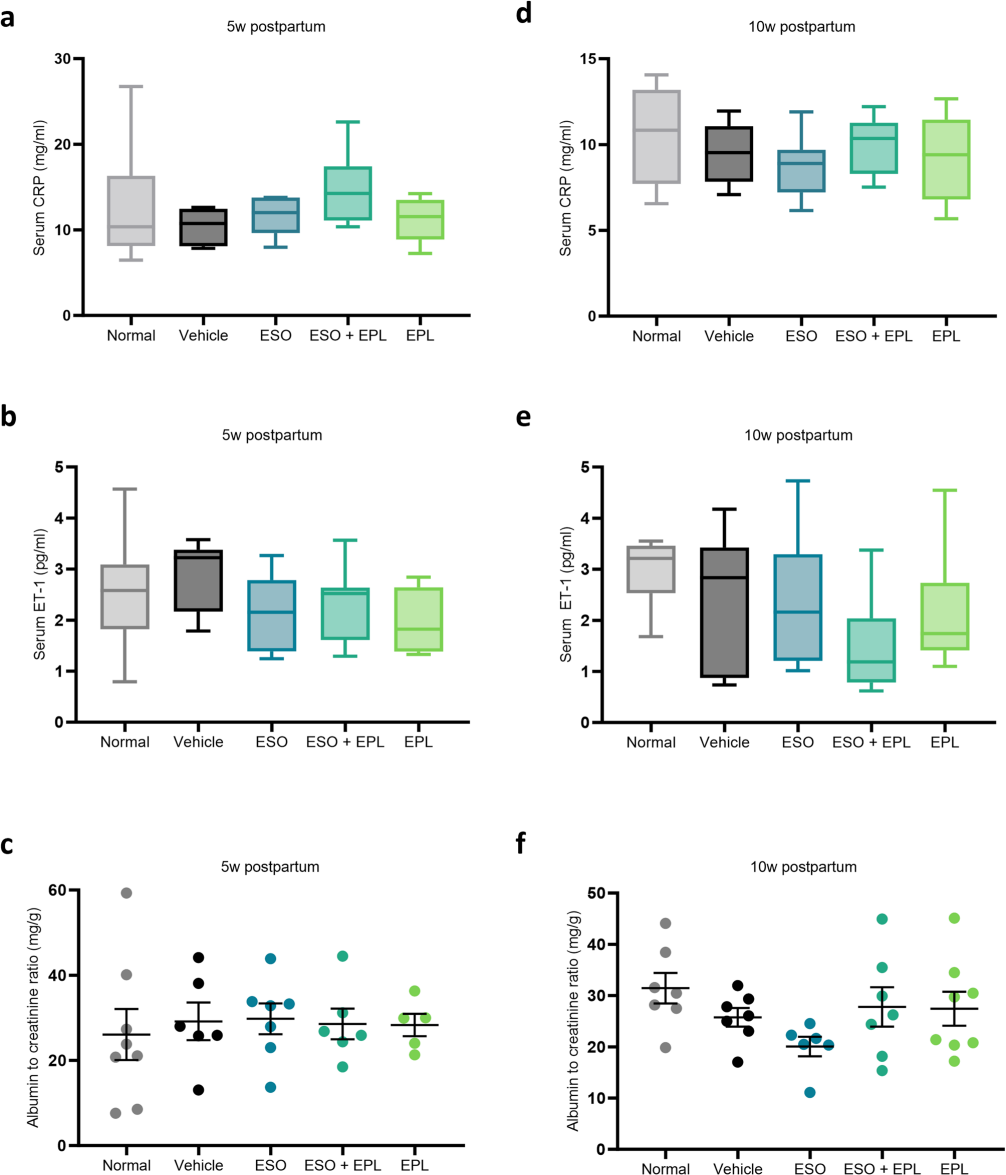
Serum and urine markers of cardiovascular and renal function did not change. At both 5 and 10 weeks postpartum, serum concentrations of CRP (**a**,**b**, respectively) and ET-1 (**d**,**e**, respectively) were not significantly different between mice that had a normal pregnancy (normal) and mice that had a preeclampsia-like pregnancy (vehicle). Similarly, treatment with esomeprazole alone (ESO), eplerenone alone (EPL), or sequential esomeprazole and eplerenone (ESO + EPL) did not affect serum CRP or ET-1 concentrations at either time point. Urine albumin to creatinine ratio at both 5 and 10 weeks postpartum was not significantly different between mice that had a normal pregnancy and mice that had a preeclampsia-like pregnancy (**c**,**f**, respectively). Similarly, treatment with ESO alone, EPL alone, or sequential ESO + EPL did not affect urine albumin to creatinine ratio. Data are mean ± SEM, *n* = 7–8.

Serum concentrations of CRP and ET-1 at 5 and 10 weeks postpartum were not significantly different between mice that had a normal pregnancy and mice that had a preeclampsia-like pregnancy (Fig. 5). Similarly, treatment with ESO, EPL, or sequential ESO + EPL did not affect serum CRP or ET-1 concentrations at either postpartum timepoints.

### A preeclampsia-like pregnancy was not associated with postpartum proteinuria

Urine albumin to creatinine ratio at both 5 and 10 weeks postpartum was not significantly different between mice that had a normal pregnancy and mice that had a preeclampsia-like pregnancy (Fig. 5). Similarly, treatment with ESO, EPL, or sequential ESO + EPL did not modulate urine albumin to creatinine ratio at either postpartum timepoint.

### Markers of cardiac remodelling were not altered in the postpartum period following a preeclampsia-like pregnancy

Cardiac gene expression of *Vcam*,* Edn1*,* Ccr2*,* Ctgf*,* Tgfb1*,* Tgfb2*,* Tgfb3*,* Bnp*,* Cybb*,* Mmp2*,* Mmp9*,* Timp1*,* Camk2a*,* Slc9a1*,* Nr3c2*, and *Nr3c1* was not different between mice who had a normal pregnancy and mice who had a preeclampsia-like pregnancy at 5 or 10 weeks postpartum (Supplementary Figs. 3 and 4, respectively). Similarly, there was no further regulation by ESO or EPL on these targets. Cardiac tissue macrophage number and collagen content assessed at 5 and 10 weeks postpartum by immunostaining and Sirius red staining, respectively, showed no differences between the groups (Supplementary Figs. 7 and 8).

### Postpartum renal gene expression was not altered following a preeclampsia-like pregnancy

 Renal gene expression of *Hsd11b2*,* Sgk1*,* Scnn1a*,* Nox4*, and *Fn1* was not different between mice who had a normal pregnancy and mice who had a preeclampsia-like pregnancy at 5 or 10 weeks postpartum (Supplementary Figs. 5 and 6, respectively). Similarly, there was no further regulation by ESO or EPL on these targets. Macrophage number in renal tissue at 5 and 10 weeks were assessed and revealed no differences between groups (Supplementary Fig. 9).

## Discussion

Preeclampsia remains one of the most significant complications of pregnancy, contributing to high global rates of maternal and neonatal death. Research has largely failed to consider interventions that target the long-term cardiovascular sequelae that follows preeclampsia^12–16^. Using L-NAME to induce a preeclampsia-like phenotype in mice, we assessed the ability of ESO during pregnancy and EPL in the postpartum period to improve measures of postpartum cardiovascular health.

L-NAME is a potent and selective inhibitor of nitric oxide synthase, causing systemic vasoconstriction due to reduced bioavailable nitric oxide, a molecule essential for vasodilation. We have previously demonstrated that L-NAME induces a preeclampsia-like phenotype in pregnant mice, including increased blood pressure, fetal growth restriction, and elevated circulating levels of sFLT1, ET-1, and CRP^48,51,52^. In this study, mice experiencing an L-NAME-induced, preeclampsia-like pregnancy had significantly increased mean arterial blood pressure on E17.5 of pregnancy and significantly decreased pup birth weight, neither of which was rescued with ESO treatment. Mice who had a preeclampsia-like pregnancy showed significantly increased vasoconstriction to one dose of phenylephrine at 5 weeks postpartum, when compared to mice who had a normal pregnancy. Postpartum EPL treatment (with or without ESO treatment during pregnancy) in mice who had a preeclampsia-like pregnancy returned postpartum vasoconstriction to normal levels. The vascular response to EPL was also associated with significant increases in postpartum vasorelaxation.

Postpartum vascular activity was measured in mesenteric arteries to assess systemic vascular adaptations following a preeclampsia-like pregnancy. Mesenteric arteries are resistance arteries involved in blood pressure regulation^56,57^. It has been hypothesised that persistent endothelial and vascular dysfunction following preeclampsia contributes to increased cardiovascular risk. Here, we showed increased ex vivo vasoconstriction of mesenteric arteries 5 weeks postpartum from mice that had a preeclampsia-like pregnancy. Importantly, EPL treatment (with or without ESO) significantly reduced vasoconstriction, to levels equivalent to mice that had a normal pregnancy. By 10 weeks postpartum, vascular function had recovered. Of note, 5 and 10 weeks postpartum in this age group of mice is approximately equivalent to 5 and 10 years postpartum in humans^58^. Following a pregnancy complicated by preeclampsia in women, there is a 3- to 4-fold increased risk of heart attack and stroke from as early as 10 years postpartum and that level of risk is maintained for up to 20 years postpartum^59^. Future studies examining later postpartum time points as well as therapeutic treatment in uncomplicated pregnancies would provide further support for the vascular reactivity findings described here. Interestingly, EPL increased vasorelaxation at 5 and 10 weeks postpartum (despite vasorelaxation not being different following a preeclampsia-like pregnancy), suggesting an independent role for MR signalling in the vessel wall, potentially in endothelial cells^36^. Improved vascular reactivity with EPL postpartum may reduce cardiovascular risk.

This model has demonstrated that even without development of overt postpartum cardiovascular changes, such as elevated blood pressure, vascular adaptations exist post-preeclampsia. While the model perhaps did not recapitulate the severity of disease seen in humans, with AUC analysis not revealing broad dysfunction in vasoconstriction or vasorelaxation, it does suggest that even a mild elevation of blood pressure during pregnancy may confer increased lifelong risk of cardiovascular disease, which EPL may mitigate. This is in line with the disproportionate risk of cardiovascular disease associated with small increases in blood pressure in the general population^60^. It is not yet clear whether having preeclampsia causes such extensive damage to the pregnant woman’s endothelium that other diseases involving endothelial dysfunction subsequently ensue^61^, or whether similarities in disease pathophysiology between preeclampsia and cardiovascular disease originate from a common genetic predisposition^62^. Certainly, the increased vasoconstriction observed at 5 weeks postpartum indicates that endothelial and vascular damage from a preeclampsia-like pregnancy persists postpartum, while at 10 weeks postpartum in the mouse this was no longer significantly apparent. EPL has well documented protective effects on the endothelial and vascular smooth muscle cells of the vessel wall in models of obesity induced heart disease in both male and female animals^63,64^, and has been shown to ameliorate adverse cardiac effects of ageing^65^. Improvements in vascular reactivity with EPL in the present study are consistent with a role for the MR in preeclampsia-induced vascular dysfunction. Given that EPL is a selective MR antagonist, unlike spironolactone which can modulate sex hormone receptors, it may have utility in modulating cardiovascular risk postpartum in women with preeclamptic pregnancies. However, data is relatively limited so far. These data suggest that development of preeclampsia therapeutics that treat endothelial and vascular dysfunction, including MR antagonists, are likely to also reduce postpartum cardiovascular risk^63,64^.

To test this hypothesis, mice receiving L-NAME to induce a preeclampsia-like phenotype also received ESO during pregnancy. We have previously demonstrated a profound improvement in preeclamptic disease characteristics in a placenta-specific sFLT1 overexpression mouse model^44^, so much so that ESO progressed to clinical trial for the treatment of preeclampsia^46,66^. Surprisingly, in this model, ESO was unable to significantly decrease blood pressure during pregnancy and did not rescue fetal growth restriction. Since completing this study, we have conducted further investigation of ESO in a nitric oxide synthase antagonist model, and believe that some of its function may be occurring through the nitric oxide pathway^52^, such that ESO is unable to overcome the potent inhibition of the pathway caused by L-NAME. Despite widespread use of ESO (proton pump inhibitor) for the treatment of gastric reflux, the extent of ESO’s off-target actions in the setting of preeclampsia remains unclear.

Mild vasoactivity changes and the lack of other postpartum indices of cardiovascular risk, such as elevated blood pressure, cardiac and renal damage, and increased circulating ET-1 and CRP in this model suggests that the detrimental effects of preeclampsia may extend beyond persistent endothelial dysfunction, and quite possibly include a genetic predisposition in humans. Following exposure to a preeclamptic pregnancy and other hypertensive disorders of pregnancy, blood pressure can remain elevated for as long as 20 years postpartum^67^, yet we observed no postpartum changes in blood pressure in this model. We also observed no change in the expression of genes associated with cardiac and renal injury, and no evidence of proteinuria, in contrast to reports of postpartum proteinuria in women following preeclampsia^68–70^. Similarly, serum ET-1 and CRP were not elevated at either 5 or 10 weeks postpartum, despite both being significantly upregulated during L-NAME-induced, preeclampsia-like pregnancy^51^. ET-1 is a potent vasoconstrictor and marker of endothelial dysfunction, while CRP is released into circulation in response to inflammation and is a clinically relevant marker of cardiovascular disease risk. Significantly, the risk of developing preeclampsia in a second pregnancy can be as high as 55%^71^ and increases even further for a third pregnancy^72^. This may reflect a ‘priming’ or changed response to repeat exposure. Perhaps exposure to a second preeclampsia-like pregnancy in this model will better recapitulate the severity of postpartum cardiovascular disease seen in humans. Importantly, one strength of the current model is our consideration of maternal reproductive lifespan, with dam age selected to support human equivalent reproductive stages^58^. Additionally, in the postpartum period, our primary focus is on the time since pregnancy rather than absolute maternal age, as our interest lies in postpartum adaptations driven by prior gestational risks rather than age-related differences within this relatively restricted age range.

## Conclusion

Despite ongoing interest in developing treatments for preeclampsia, there remains an urgent unmet need for interventions focused on maternal postpartum cardiovascular health. While the results of this study suggest that postpartum therapeutics may offer some benefits in relation to improvements in vascular function after preeclampsia in the medium to longer term, the need to further develop this and other animal models investigating postpartum effects of preeclampsia is undeniable. No one animal model can completely recapitulate the gambit of preeclampsia pathophysiology, and promising therapeutics should be validated in multiple preclinical models before progressing to clinical trial. Importantly, like ESO in pregnancy, EPL has a good safety profile in younger women, with very low risk of hyperkalaemia, and no sex steroid receptor effects, unlike other MR antagonists such as spironolactone.

This study was supported by Baker Department of Cardiometabolic Health, University of Melbourne Seed Funding to NJH and MJY. Salary support was provided by the National Health and Medical Research Council Fellowships to NJH (#1146128). The funders played no role in study design or analysis. MJY is supported by Baker Trust Alice Baker and Eleanor Shaw Gender Equity Fellowship, Perpetual Philanthropy, Diabetes Australia. The Baker HDI is supported by the Victorian Government’s Operational Infrastructure Scheme.

## Electronic supplementary material

Below is the link to the electronic supplementary material.

Supplementary Material 1

## Data Availability

All relevant data of this study are available from the corresponding author upon reasonable request.
