# Supplementary material for: Postpartum administration of eplerenone to mitigate vascular dysfunction in mice following a preeclampsia-like pregnancy
Source: Sci Rep. 2025 May 27;15:18455. doi: 10.1038/s41598-025-02475-0 (PMC12116800; doi:10.1038/s41598-025-02475-0)
Supplement: Supplementary file 1 — Supplementary Material 1 [file 41598_2025_2475_MOESM1_ESM.pdf]

Supplementary figure 1

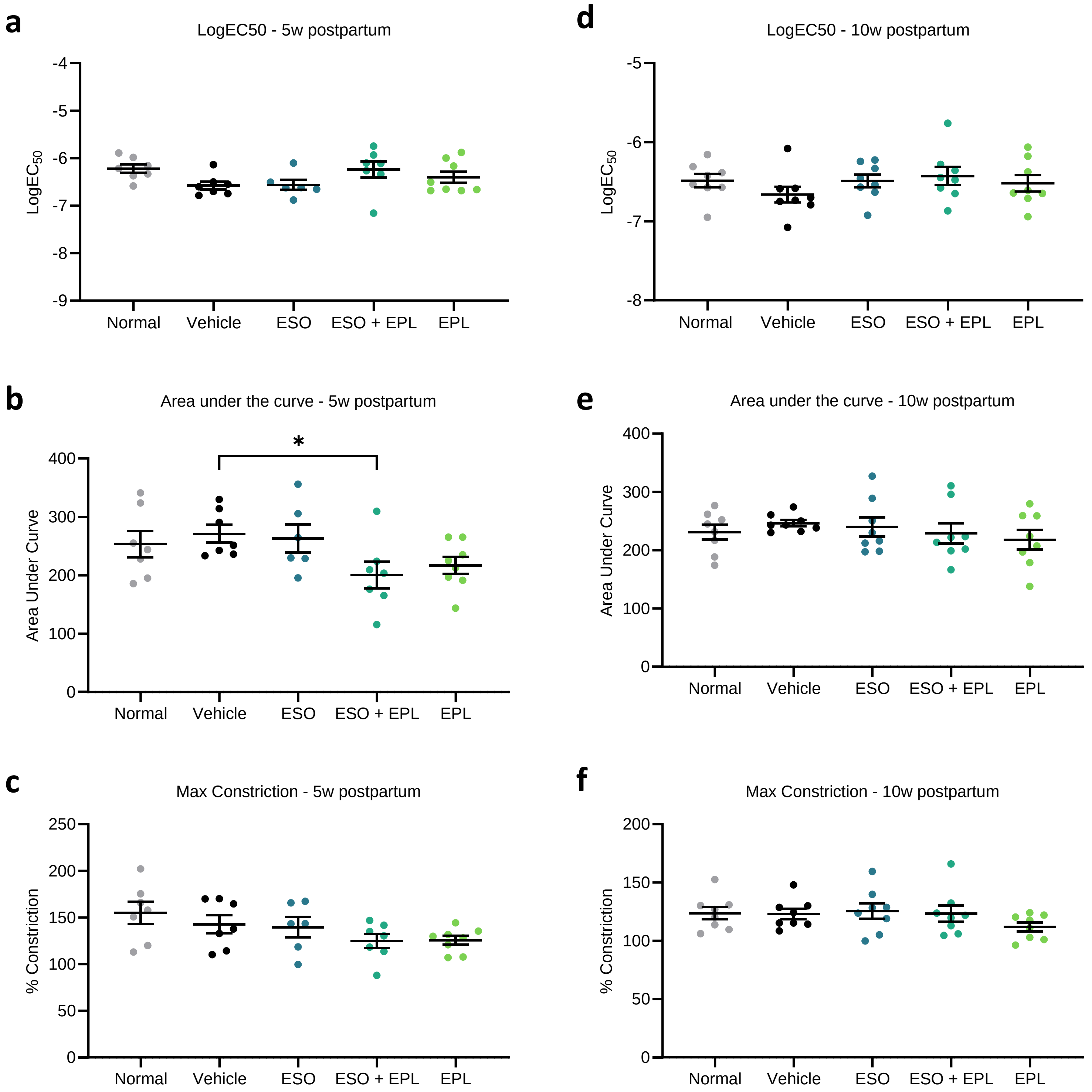

Supplementary figure 2

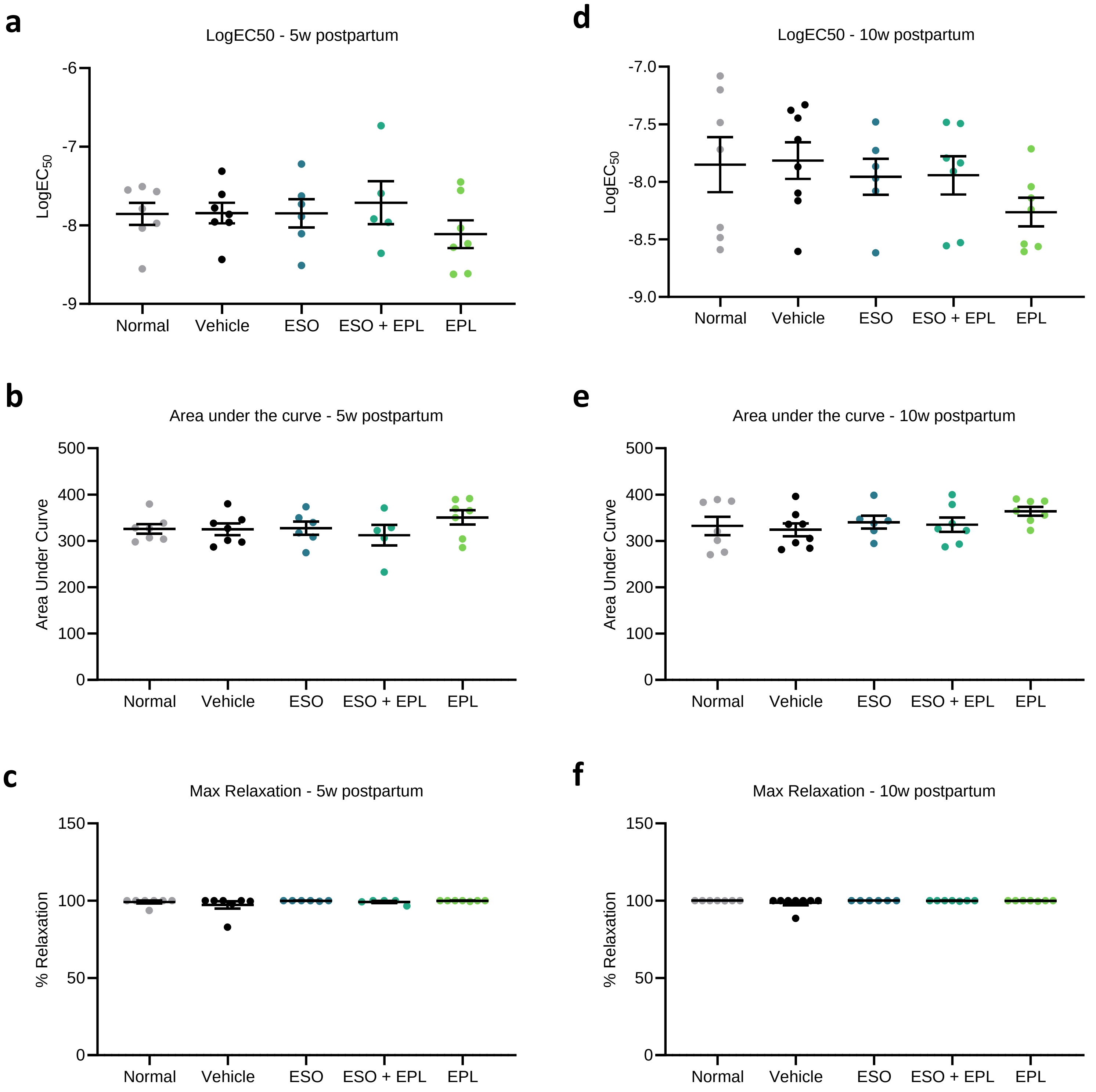

Supplementary figure 3

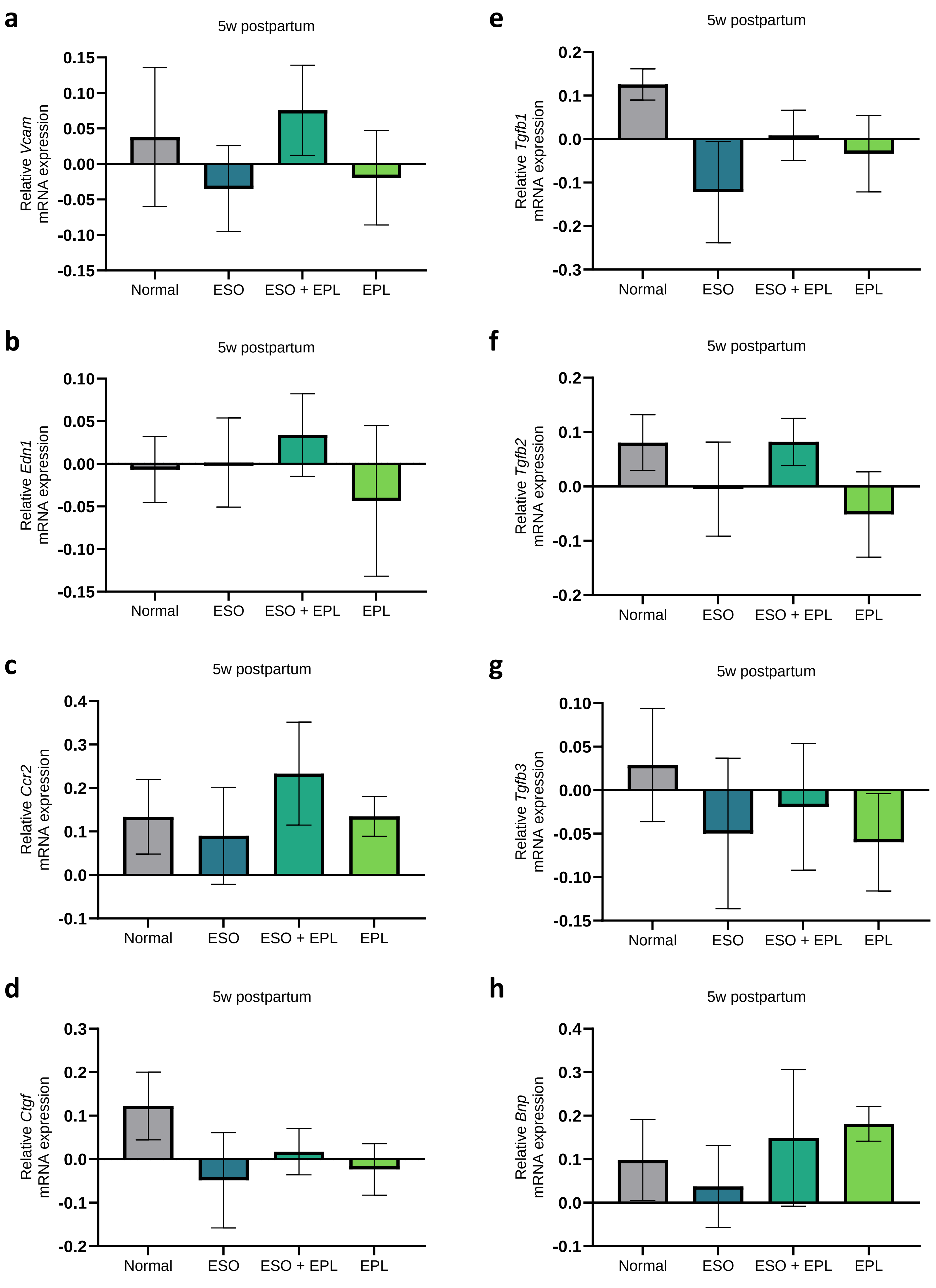

Supplementary figure 3 continued

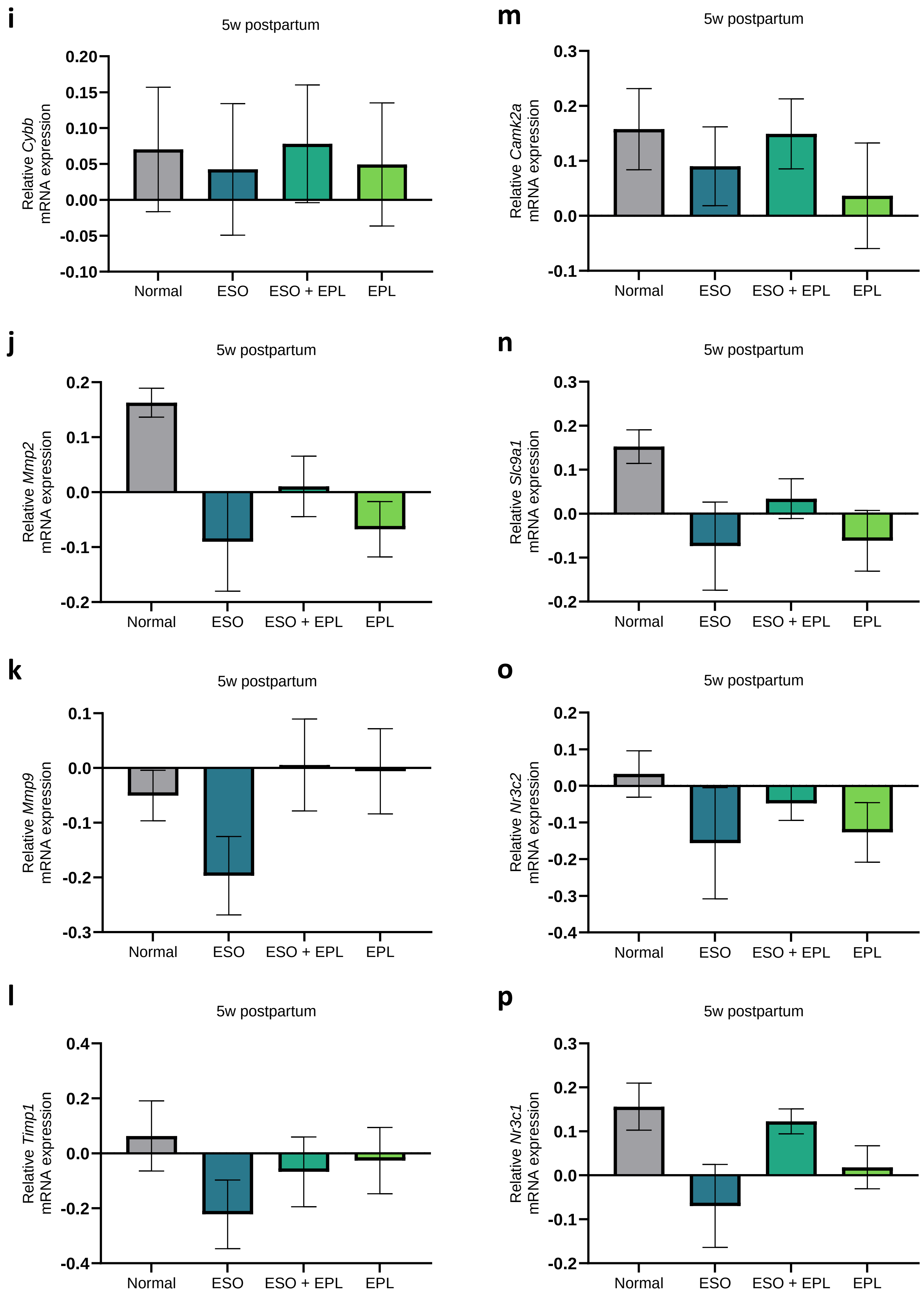

Supplementary figure 4

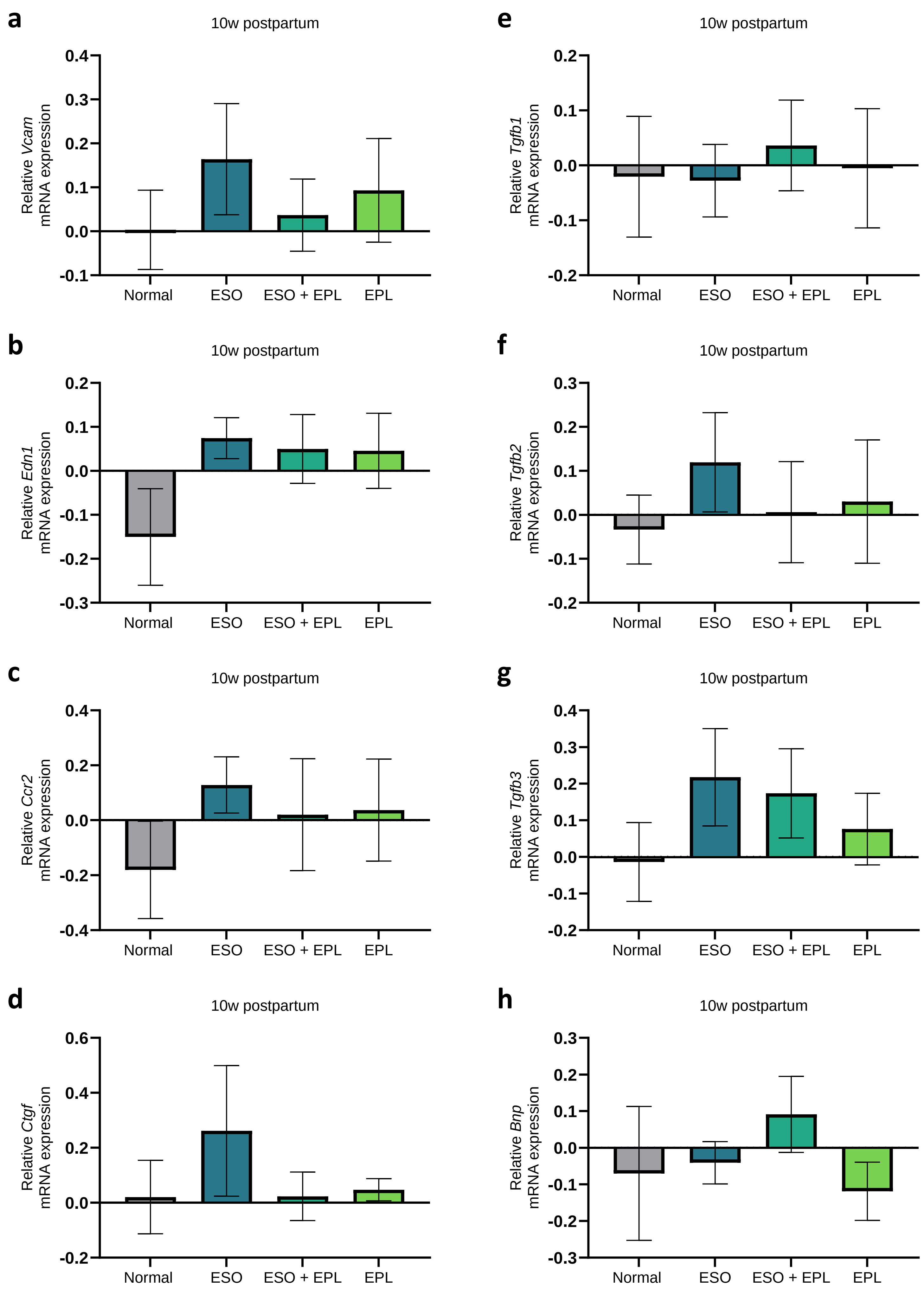

Supplementary figure 4 continued

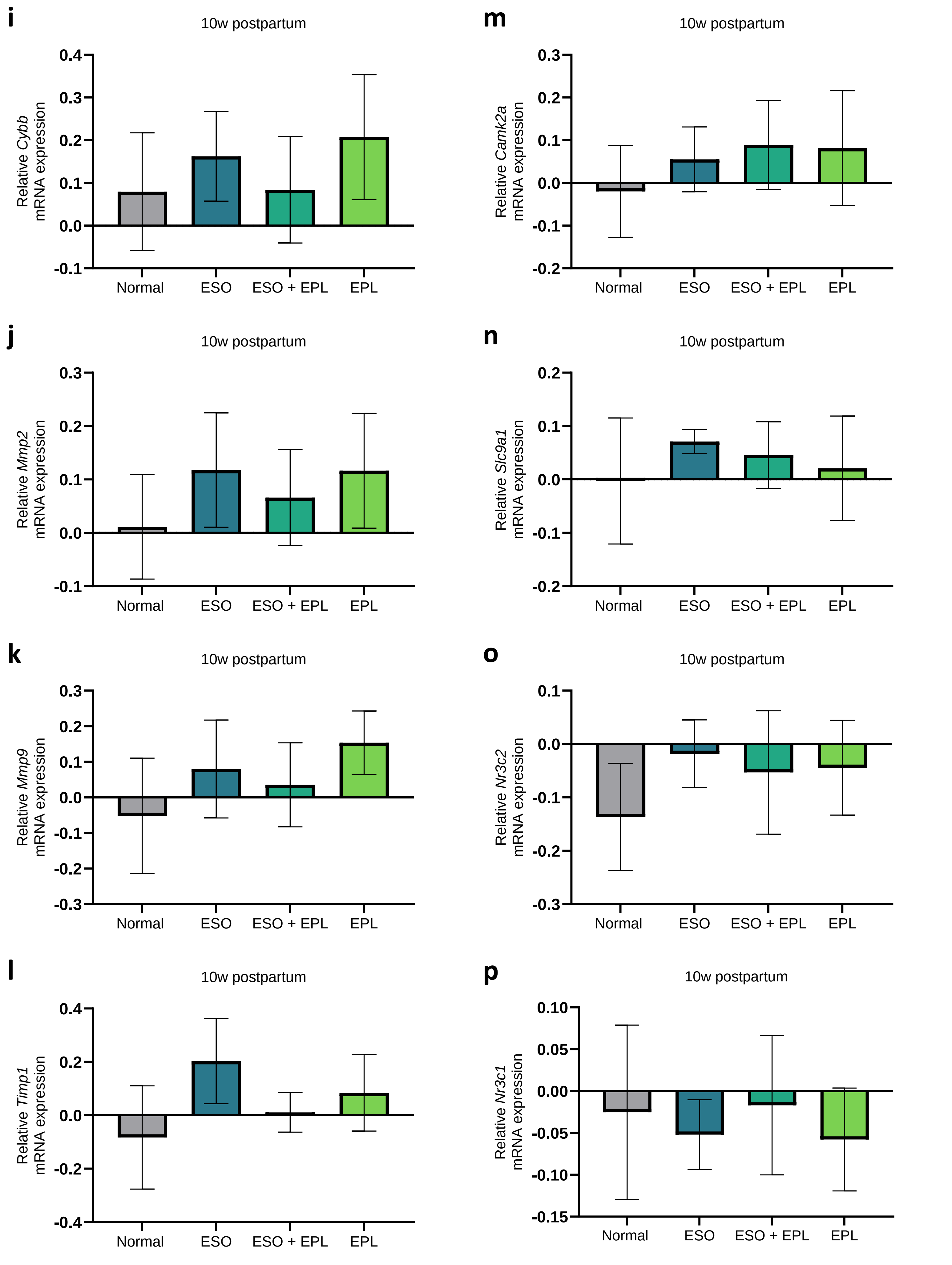

Supplementary figure 5

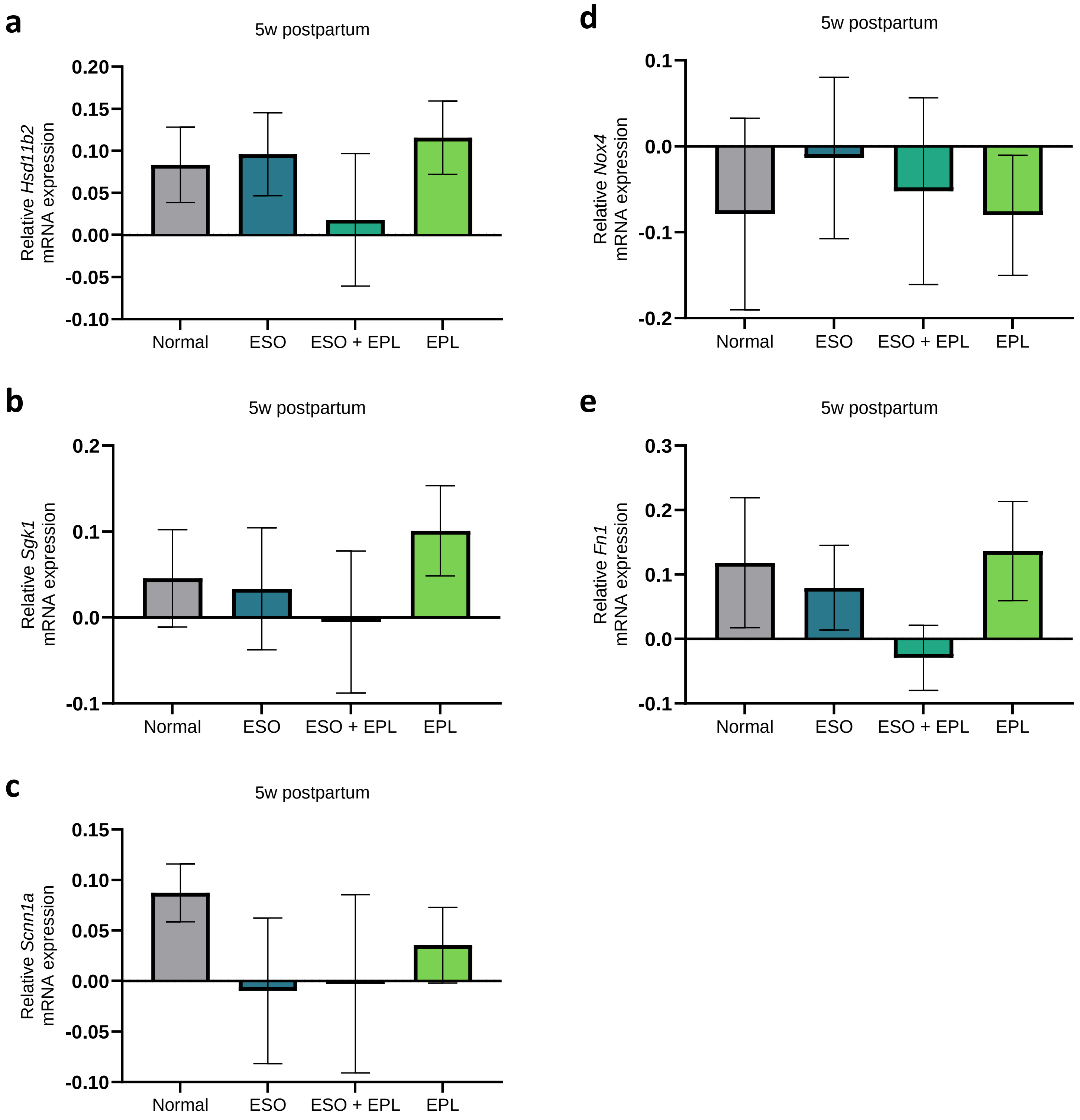

Supplementary figure 6

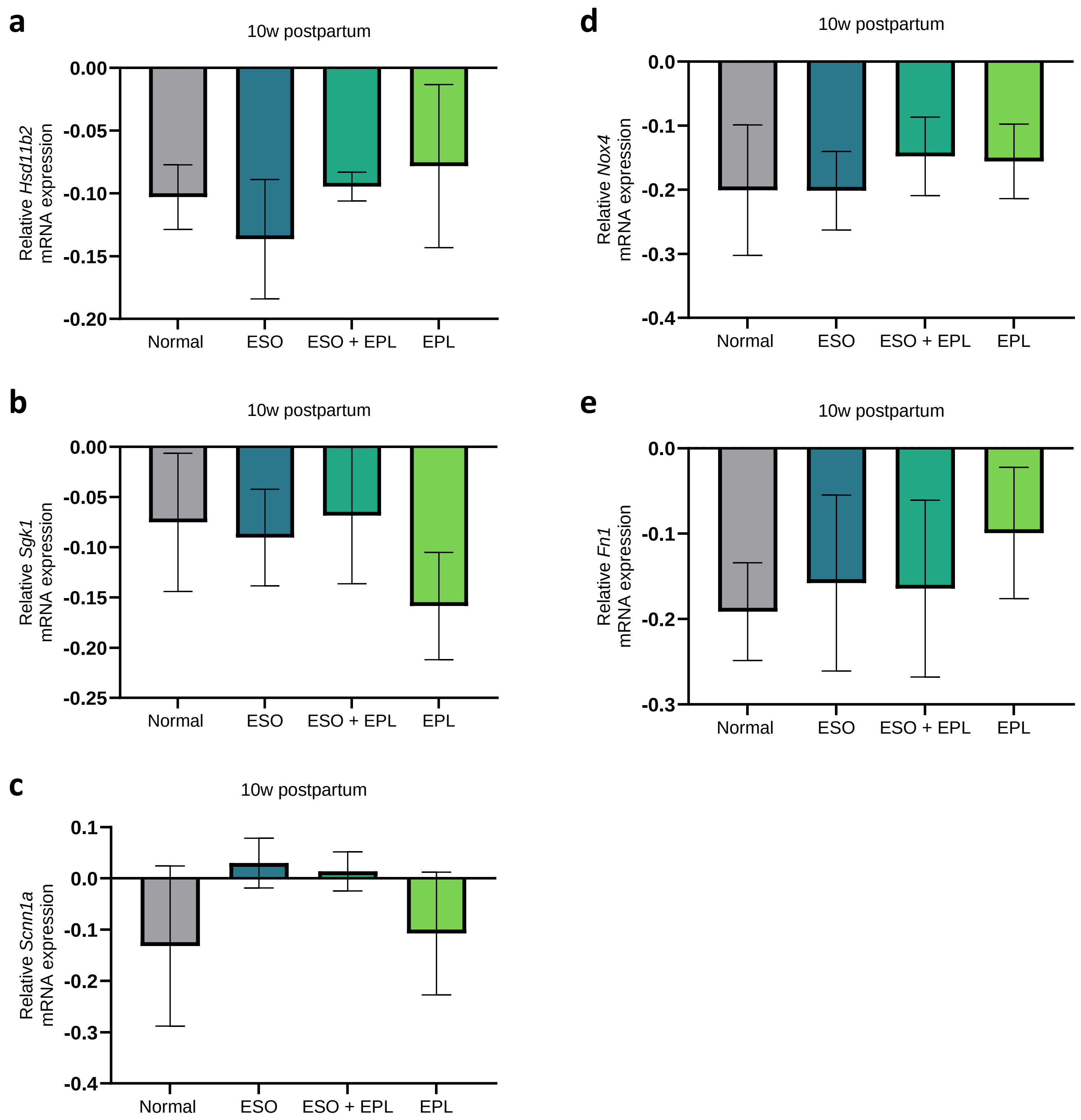

Supplementary figure 7

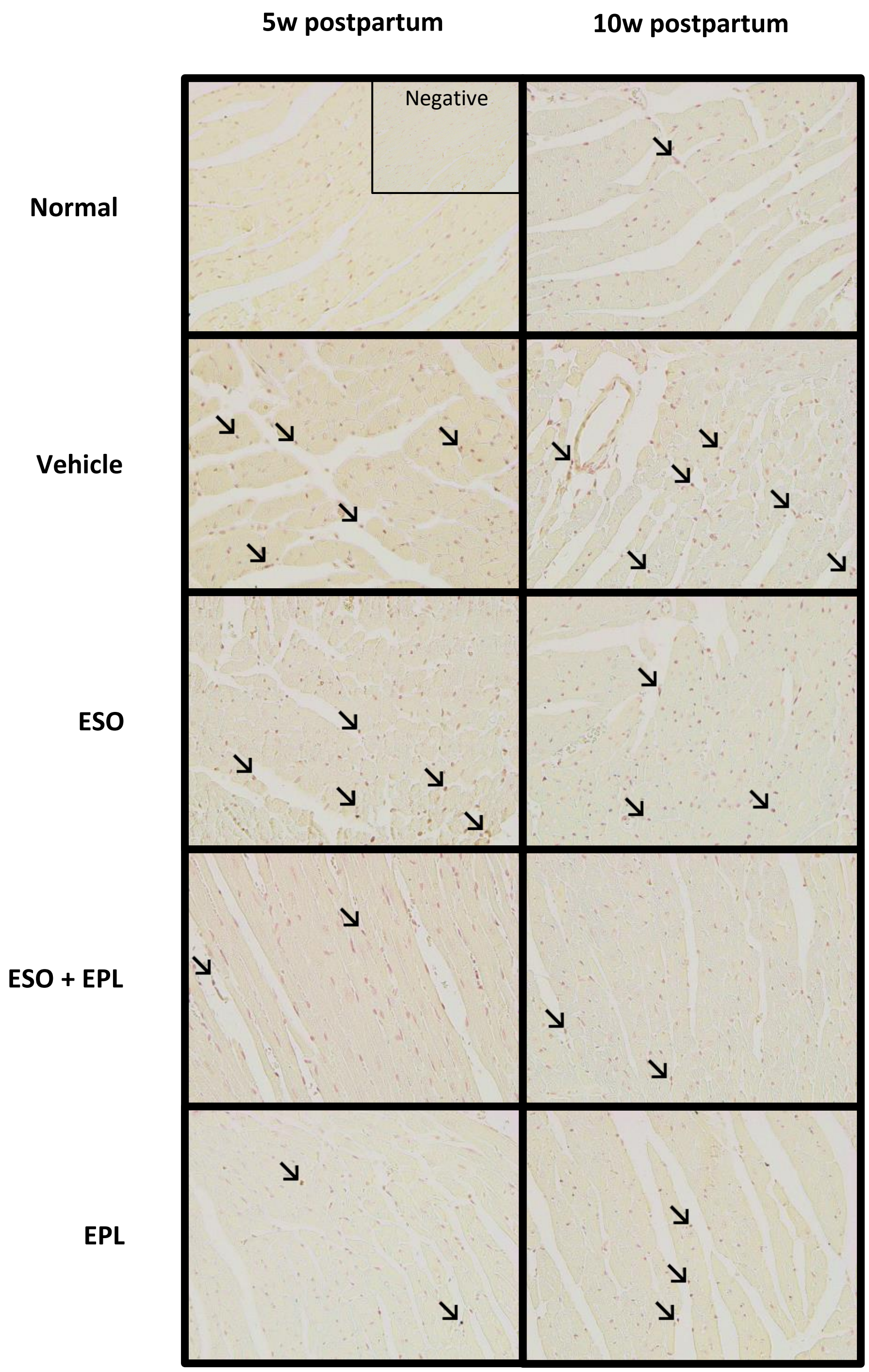

Supplementary figure 8

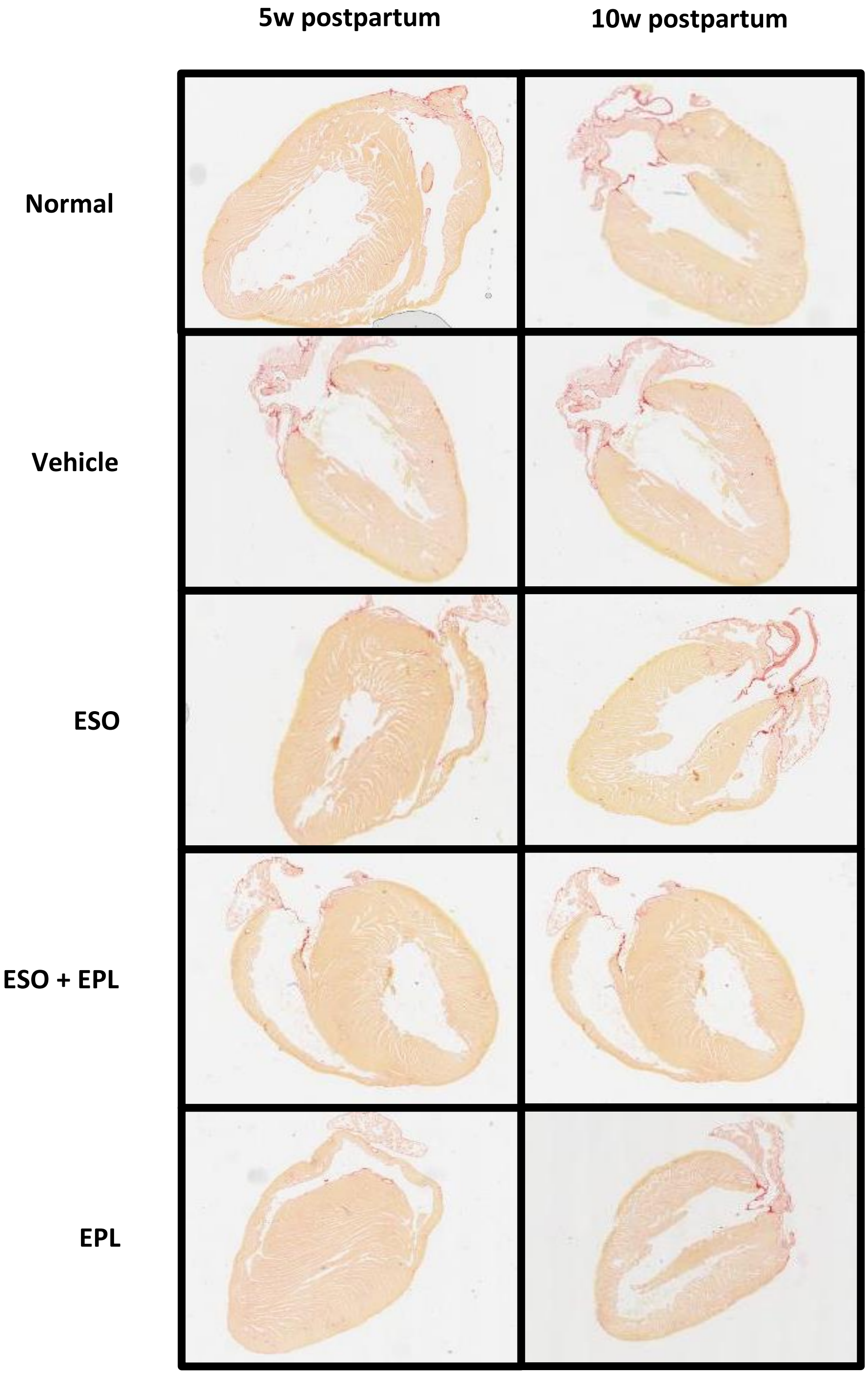

Supplementary figure 9

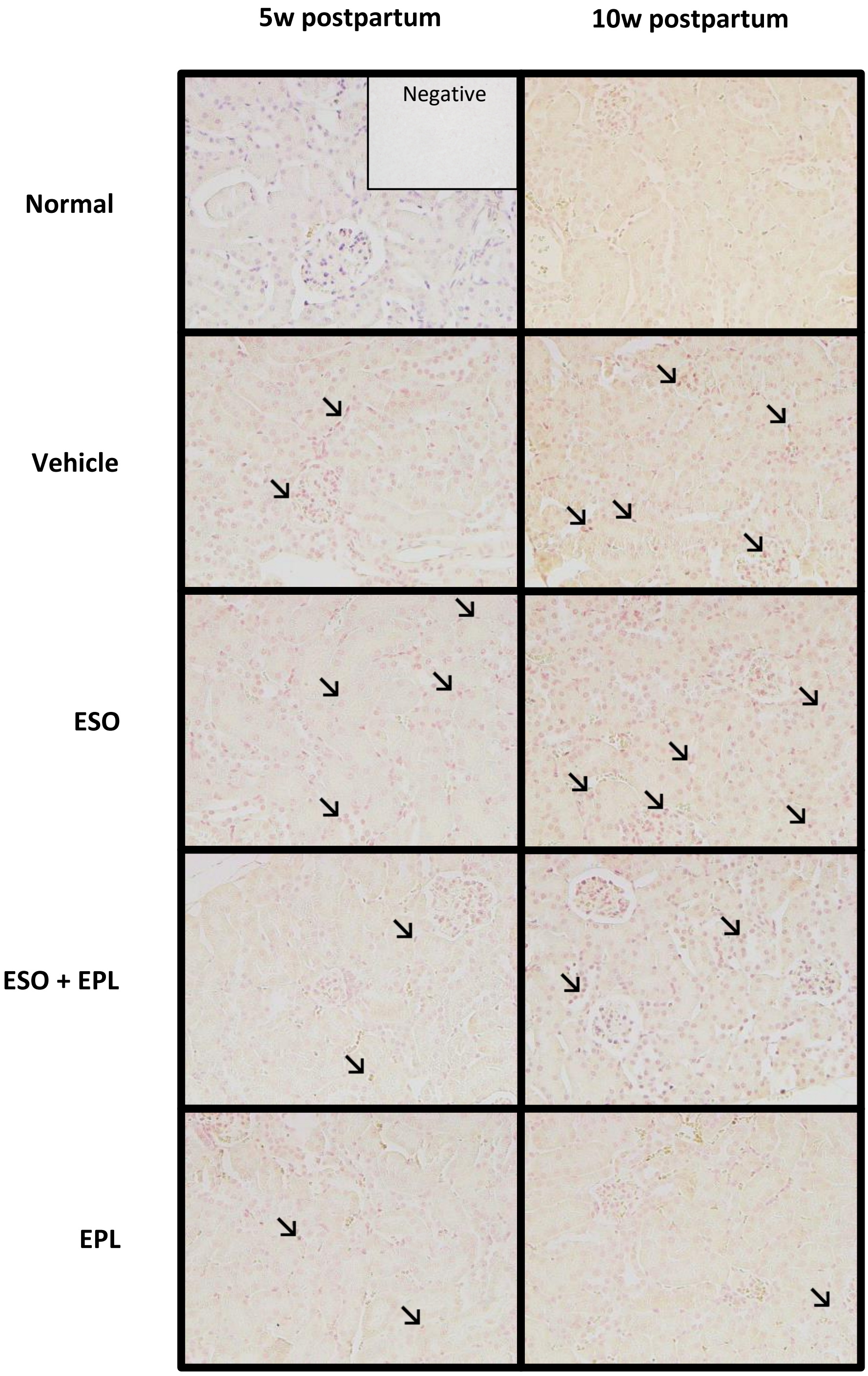

Supplementary figure legends

Supplementary figure 1: Analysis of vasoconstriction curves. At 5 weeks postpartum (b), but not 10 weeks (e), area under the curve (total response) was significantly reduced for constriction curves generated from mesenteric arteries excised from mice who received sequential esomeprazole and eplerenone (ESO + EPL) treatment. No significant changes were observed in the LogEC50 (a, d) and maximum response to vasoconstriction (c, f) at 5 and 10 weeks postpartum, respectively. Normal, normal pregnancy; vehicle, preeclampsia-like pregnancy; ESO, esomeprazole alone; EPL, eplerenone alone. Data are mean ± SEM, n=6-8, *p<0.05.

Supplementary figure 2: Analysis of vasorelaxation curves. No significant changes were observed in the LogEC50 (a, d), area under the curve (b, e), and maximum relaxation (c, f) at 5 and 10 weeks postpartum, respectively. Normal, normal pregnancy; vehicle, preeclampsia-like pregnancy; ESO, esomeprazole alone; EPL, eplerenone alone; ESO + EPL, sequential esomeprazole and eplerenone. Data are mean ± SEM, n=6-8.

Supplementary figure 3: Cardiac gene expression at 5 weeks postpartum. There was no change in cardiac expression of *Vcam* (a), *Edn1* (b), *Ccr2* (c), *Ctgf* (d), *Tgfb1* (e), *Tgfb2* (f), *Tgfb3* (g), *Bnp* (h), *Cybb* (i), *Mmp2* (j), *Mmp9* (k), *Timp1* (l), *Camk2a* (m), *Slc9a1* (n), *Nr3c2* (o), and *Nr3c1* (p) between mice who had a normal pregnancy and mice who had a preeclampsia-like pregnancy at 5 weeks postpartum. Similarly, there was no further regulation by esomeprazole alone (ESO), eplerenone alone (EPL), or sequential esomeprazole and eplerenone (ESO + EPL) on these targets. Data are normalised to samples from mice who had a preeclampsia-like pregnancy (baseline expression at 0.0) and log transformed (above 0.0, increased expression; below 0.0, decreased expression) ± SEM, n=4.

Supplementary figure 4: Cardiac gene expression at 10 weeks postpartum. There was no change in cardiac expression of *Vcam* (a), *Edn1* (b), *Ccr2* (c), *Ctgf* (d), *Tgfb1* (e), *Tgfb2* (f), *Tgfb3* (g), *Bnp* (h), *Cybb* (i), *Mmp2* (j), *Mmp9* (k), *Timp1* (l), *Camk2a* (m), *Slc9a1* (n), *Nr3c2* (o), and *Nr3c1* (p) between mice who had a normal pregnancy and mice who had a preeclampsia-like pregnancy at 10 weeks postpartum. Similarly, there was no further regulation by esomeprazole alone (ESO), eplerenone alone (EPL), or sequential esomeprazole and eplerenone (ESO + EPL) on these targets. Data are normalised to samples from mice who had a preeclampsia-like pregnancy (baseline expression at 0.0) and log transformed (above 0.0, increased expression; below 0.0, decreased expression) ± SEM, n=4.

Supplementary figure 5: Renal gene expression at 5 weeks postpartum. There was no change in renal expression of *Hsd11b2* (a), *Sgk1* (b), *Scnn1a* (c), *Nox4* (d), and *Fn1* (e) between mice who had a normal pregnancy and mice who had a preeclampsia-like pregnancy at 5 weeks postpartum. Similarly, there was no further regulation by esomeprazole alone (ESO), eplerenone alone (EPL), or sequential esomeprazole and eplerenone (ESO + EPL) on these targets. Data are normalised to samples from mice who had a preeclampsia-like pregnancy (baseline expression at 0.0) and log transformed (above 0.0, increased expression; below 0.0, decreased expression) ± SEM, n=4.

Supplementary figure 6: Renal gene expression at 10 weeks postpartum. There was no change in renal expression of *Hsd11b2* (a), *Sgk1* (b), *Scnn1a* (c), *Nox4* (d), and *Fn1* (e) between mice who had a normal pregnancy and mice who had a preeclampsia-like pregnancy at 10 weeks postpartum. Similarly, there was no further regulation by esomeprazole alone (ESO), eplerenone alone (EPL), or sequential esomeprazole and eplerenone (ESO + EPL) on these targets. Data are normalised to samples from mice who had a preeclampsia-like pregnancy (baseline expression at 0.0) and log transformed (above 0.0, increased expression; below 0.0, decreased expression) ± SEM, n=4.

Supplementary figure 7: Cardiac inflammatory cell infiltration. Representative immunostaining for mac2 (galectin 3) for inflammatory cell infiltrate in hearts from females at either 5 weeks or 10 weeks postpartum. Arrows indicate staining. Normal, normal pregnancy; vehicle, preeclampsia-like pregnancy; ESO, esomeprazole alone; EPL, eplerenone alone; ESO + EPL, sequential esomeprazole and eplerenone.

Supplementary figure 8: Assessment of cardiac collagen networks. Representative Sirius red staining for connective tissue in hearts from females at either 5 weeks or 10 weeks postpartum. Normal, normal pregnancy; vehicle, preeclampsia-like pregnancy; ESO, esomeprazole alone; EPL, eplerenone alone; ESO + EPL, sequential esomeprazole and eplerenone.

Supplementary figure 9: Renal inflammatory cell infiltration. Representative immunostaining for mac2 (galectin 3) for inflammatory cell infiltrate in kidney from females at either 5 weeks or 10 weeks postpartum. Arrows indicate staining. Normal, normal pregnancy; vehicle, preeclampsia-like pregnancy; ESO, esomeprazole alone; EPL, eplerenone alone; ESO + EPL, sequential esomeprazole and eplerenone.
